# Assessment on the incidence of notifiable infectious diseases in pediatrics post-COVID-19: a retrospective study based on data from 2020 to 2023

**DOI:** 10.3389/fped.2025.1648443

**Published:** 2025-10-15

**Authors:** Wei Sun, Zujin Luo, Shunli Li, Hongbin Zhu, Yingmin Ma, Jing Wang

**Affiliations:** ^1^ Department of Respiratory and Critical Care Medicine, Beijing Institute of Respiratory Medicine and Beijing Chao-Yang Hospital, Capital Medical University, Beijing, China; ^2^ Department of Infection Management and Disease Control, Beijing Chaoyang Hospital, Capital Medical University, Beijing, China; ^3^ Department of Pediatrics, Beijing Chaoyang Hospital, Capital Medical University, Beijing, China; ^4^ Department of Respiratory and Critical Care Medicine, Beijing Youan Hospital, Capital Medical University, Beijing, China

**Keywords:** COVID-19, pediatrics, immunity debt, infectious diseases, influenza

## Abstract

**Background and aim:**

The Coronavirus Disease 2019 (COVID-19) pandemic has notably affected pediatric health, especially regarding notifiable infectious diseases. While strict control measures reduced infection transmission, they also lowered herd immunity, possibly causing a rise in pediatric infectious disease cases once restrictions were eased. The aim of the study is to compare the number of pediatric outpatient and emergency visits and the incidence rates of notifiable infectious diseases from 2020 to 2023.

**Methods:**

We retrospectively analyzed pediatric department visit cases from 2020 to 2023 and compared variations in the number and proportion of notifiable infectious diseases across different years.

**Results:**

Our findings revealed a sharp increase in pediatric visits, from 31,401 before the pandemic to 89,945 in 2023, representing an approximate threefold increase. Notifiable infectious disease cases rose from 224 in 2020 to 1027 in 2023, marking an increase of nearly 4.6 times. The incidence of influenza, hand-foot-mouth disease (HFMD), and infectious diarrhea also markedly increased.

**Conclusions:**

These findings establish a significant association between the relaxation of COVID-19 restrictions and increased pediatric infectious disease occurrence. In 2023, there has been a substantial increase in the number of pediatric outpatient and emergency department visits, as well as in the incidence rate of notifiable infectious diseases, when compared to the period from 2020 to 2022. Notably, the incidence of influenza has exhibited the most pronounced increase, while the incidence rates of measles and mumps have remained stable.

## Background

The Coronavirus Disease 2019 (COVID-19) pandemic has profoundly transformed the landscape of pediatric infectious diseases, prompting essential inquiries into its effects on the incidence and management of these diseases in the pediatric population. Previous studies have focused on the immediate impact of the implementation of strict public health measures during the pandemic, such as social distancing and lockdowns, which led to a significant reduction in the transmission of various infectious diseases. Our research distinctively underscores the long-term implications of decreased community immunity due to reduced exposure to common pathogens, which leads to a heightened prevalence of infectious diseases. The complexity of the infectious cases has been exacerbated by potential alterations in the immune system induced by COVID-19 infection (1, 2).

The aim of his study is to analyze pediatric outpatient and emergency department visits, and compared the incidence rate of notifiable infectious diseases (including influenza, mumps, hand-foot-mouth disease, chickenpox and infectious diarrhea) from 2020 to 2023. This study is expected to explore how the COVID-19 pandemic has influenced the epidemiology of pediatric notifiable infectious diseases.

## Methods

### Data collection

This research is designed as a cross-sectional study and it was performed at the Department of Pediatrics of Beijing Chao-Yang Hospital Western Branch. The physician formulates a diagnosis by evaluating the child's symptoms, clinical signs, laboratory test results, and epidemiological history. In cases where the condition is classified as a legally notifiable infectious disease, the physician is required to report it through the hospital's comprehensive public health monitoring and management system, which subsequently transmits the information to the China Information System for Disease Control and Prevention (http://www.chinacdc.cn/), which is responsible for reviewing the data to eliminate false reports and duplicate cases, conducting verification and confirmation processes, and ensuring long-term storage of the information within the system. Notifiable infectious diseases are those reported in compliance with the “Law of the People's Republic of China on the Prevention and Control of Infectious Diseases.” The data pertaining to the frequency of outpatient and emergency pediatric visits from 2020 to 2023, along with the demographic characteristics and diagnoses of the patients, have been compiled and summarized by the information department of our hospital. All febrile patients underwent nucleic acid testing of nasal and pharyngeal swab specimens to rule out COVID-19. The study was approved by the Ethics Committee of Beijing Chao-Yang Hospital, which waived the requirement for informed consent.

### Statistical analysis

The proportion of notifiable infectious diseases was the absolute number of cases divided by the total number of visits. Age was represented as median values and 25% and 75% quartiles. We employ the chi-square test or Fisher's exact test to analyze the differences in gender, age group, and infectious disease incidence subgroups across various years. Statistical significance was defined as a two-tailed *P* < 0.05. All analyses were performed with SPSS Statistics for Windows version 25.0 (IBM).

## Results

**Figure 1:**
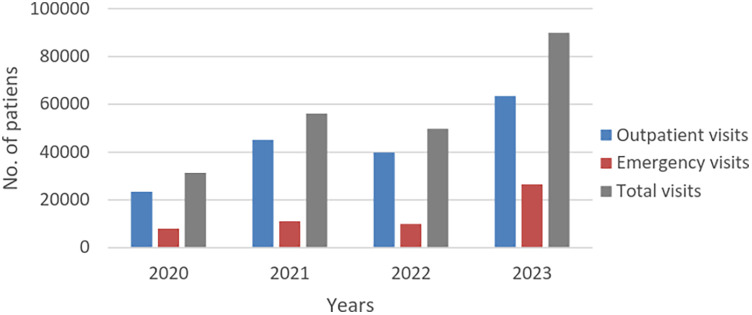
Number of outpatients and emergency pediatric patients from 2020 to 2023 (in 2023, there is a marked increase in the number of pediatric outpatient visits, emergency visits, and total visits compared to the period from 2020 to 2022).

In 2020, there were 23,385 outpatient visits and 8,016 emergency visits, resulting in a total of 31,401 visits. In 2021, the numbers increased to 45,104 outpatient visits and 11,027 emergency visits, culminating in 56,131 visits. By 2022, there were 39,876 outpatient visits and 9,922 emergency visits, with a total of 49,798 visits. By 2023, the numbers further escalated to 63,447 outpatient visits and 26,498 emergency visits, amounting to 89,945 visits (Figure 1 and Table 1).

**Table 1:** Characteristics of pediatric patients with notifiable infectious diseases.

Characteristics	2020	2021	2022	2023	*χ* ^2^	*P* value
No. of outpatient visits	23,385	45,104	39,876	63,447		
No. of emergency visits	8016	11,027	9922	26,498		
No. of total visits	31,401	56,131	49,798	89,945		
No. of notifiable infectious diseases (%)	224 (0.71)	100 (0.18)	50 (0.1)	1,027 (1.14)	802	<0.001^a^
Sex, No. (%)						
Male	121 (54)	52 (52)	29 (58)	578 (56)	1.059	0.787^b^
Female	103 (46)	48 (48)	21 (42)	449 (44)		
Age, median (IQR), y	5 (3–8)	5 (3–9)	5 (3–8)	7 (4–10)		
Age distribution, No. (%)						
<1	2 (0.9)	1 (1)	1 (2)	0 (0)	11.039	0.009^c^
≥1 < 5	96 (42.9)	40 (40)	23 (46)	316 (30.8)	19.319	<0.00^d^
≥5 < 10	90 (40.2)	39 (39)	18 (36)	410 (40)	2.518	0.474^d^
≥10 < 14	36 (16.1)	20 (20)	8 (16)	301 (29.2)	19.832	<0.001^d^
Notifiable infectious disease, No. (%)						
Influenza	171 (76.3)	3 (3)	14 (28)	734 (71.5)	232	<0.00^e^
Mumps	13 (5.8)	18 (18)	12 (24)	12 (1.2)	128	<0.00^f^
HFMD	14 (6.3)	45 (45)	11 (22)	241 (23.5)	64	<0.00^f^
Chickenpox	26 (11.6)	32 (32)	12 (24)	18 (1.8)	185	<0.00^f^
Infectious diarrhea	0(0)	2(2)	1(2)	7(0.68)	5.5	0.4^e^

IQR, interquartile range; HFMD, hand-foot-mouth disease.

^a^ *χ*^2^ test was used to compute the distribution of the incidence of notifiable infectious diseases in a 2 × 4 contingency table, comparing data from the years 2020 to 2023.

^b^ *χ*^2^ test was used to compute the distribution of the gender in a 2 × 4 contingency table, comparing data from the years 2020 to 2023.

^c^ Fisher's exact test was used to compute the distribution incidence of notifiable infectious diseases in children under one year old in a 2 × 4 contingency table, comparing data from the years 2020 to 2023.

^d^ *χ*^2^ test was used to compute the distribution of the incidence of notifiable infectious diseases in children aged 1–5, 5–10 and 10–14 in a 2 × 4 contingency table, comparing data from the years 2020 to 2023.

^e^ Fisher's exact test was used to compute the distribution incidence of influenza and Infectious diarrhea in a 2 × 4 contingency table, comparing data from the years 2020 to 2023.

^f^ Fisher's exact test was used to compute the distribution incidence of mumps, HFMD and chickenpox in a 2 × 4 contingency table, comparing data from the years 2020 to 2023.

**Figure 2:**
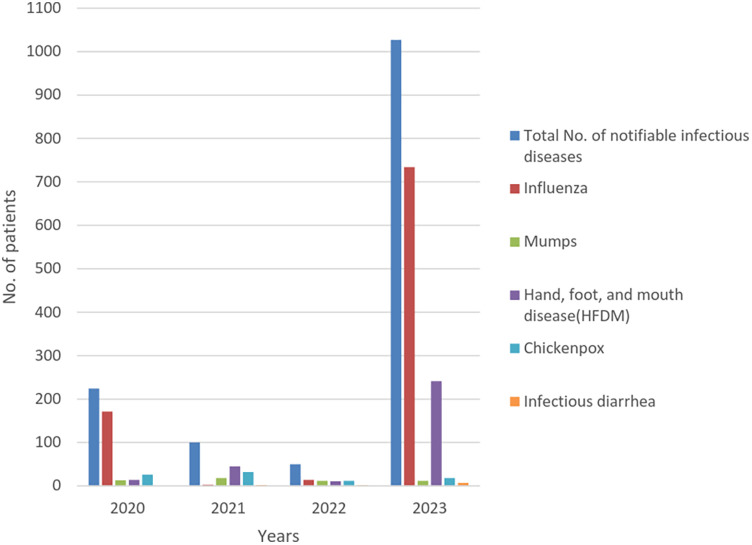
Number of total notifiable infectious diseases, influenza, mumps, HFMD, chickenpox and infectious diarrhea from 2020 to 2023 (in 2023, there is a significant increase in the incidence of influenza, HFMD and infectious diarrhea compared to the period from 2020 to 2022, whereas the incidence of mumps and chickenpox is relatively stable.) HFMD, hand-foot-mouth disease.

The incidence of notifiable infectious diseases were systematically recorded (case of notifiable infectious diseases were incorporated into the overall visitation count), revealing case counts of 224 (0.71%), 100 (0.18%), 50 (0.1%), and 1,027 (1.14%) from 2020 to 2023, respectively. Notably, the number of notifiable infectious disease cases in 2023 exhibited a substantial increase compared to the preceding years of 2020, 2021, and 2022 (*P* < 0.001) (Figure 2 and Table 1). From 2020 to 2023, there were no differences in the incidence of diseases according to sex. In 2023, the proportion of children aged 0–5 years was the lowest, whereas that of children aged 10–14 years was the highest. Furthermore, there was no significant difference in the proportion of children aged 5–10 years when comparing the data from 2020 to 2023.

The occurrence of influenza was the highest in 2023, and the incidence of influenza among patients was markedly greater in 2023 than in 2021 and 2022 (*P* < 0.001). The number of HFMD cases in 2023 exceeded those in 2020, 2021, and 2022, but the incidence of HFMD in 2023 was lower than that observed in 2021. The number of cases of infectious diarrhea in 2023 surpassed those observed in 2020, 2021, and 2022. The incidence rates of mumps and chickenpox in 2023 were lower than those recorded in previous years.

## Discussion

The current study revealed a significant rise in the incident rate of pediatric notifiable infectious diseases, outpatient visits and emergency visits and in 2023 compared to 2020–2022, with influenza seeing the largest increase, while measles and mumps rates stayed stable.

The implementation of stringent public health nonpharmacological interventions (NPI) targeting COVID-19 has been successful. However, a lack of immune stimulation due to personal NPI induces an “immunity debt,” (following a period of minimal pathogen exposure, the number of susceptible individuals increases, heightening the risk of severe epidemics when the pathogens resurface.) which could have negative consequences when the pandemic is under control (3). Our study indicates a significant resurgence in pediatric notifiable infectious diseases, especially influenza, hand-foot-mouth disease, and infectious diarrhea, highlighting the need for enhanced surveillance and tailored public health strategies in the post-COVID era.

Upsurge of pediatric infectious diseases could be attributed to the immune debt caused by pandemic-related restrictions. In New Zealand, lockdown measures led to a significant drop in infant respiratory illness hospitalizations, with no winter peak from January 1 to August 31, 2020 (4). However, following the partial easing of New Zealand's strict border closure in April 2021, RSV cases and bronchiolitis admissions surged. By week 28 of 2021, RSV cases were over five times the 2015–19 peak average (5). It was demonstrated that the decline in RSV antibodies was significant among children during the 2021 and 2023 pandemic years (6). In Germany, Christian et al. observed a decline in non-COVID-19 respiratory and gastrointestinal infections during the pandemic, with pediatric practices noting significant drops in diagnoses of influenza, pneumonia, and acute sinusitis (7). In Wuhan, researchers examined parainfluenza virus (PIV) infections from 2014 to 2022, noting a sharp drop in PIV positive rates during the 2020 COVID-19 pandemic, followed by a rebound in 2021–2022 (8). Our study revealed a resurgence in cases of notifiable infectious diseases in 2023. Following the administration of the combined measles, mumps, rubella, and varicella vaccine (MMRV), protective antibodies may persist for a lifetime, thereby offering enduring immune protection for children (9), influenza virus antigens are known for their ability to mutate rapidly, which is a significant reason why previous vaccines do not provide long-lasting immunity (10). Like the influenza vaccine, the short-lived efficacy of the HFMD vaccine necessitates booster shots or new formulations to ensure continued protection against the disease. This observation aligns with our research findings, which indicate that there was no significant increase in the number of mumps and chickenpox cases; in contrast, there was a significant rise in the incidence of influenza as well as hand, foot, and mouth disease.

Several studies have determined a correlation between COVID-19 and lymphopenia. Huang's study from early COVID-19 in Wuhan found lymphopenia in 63% of hospitalized patients, rising to 85% in severe cases (11). It has been shown that SARS-CoV-2 could infect T lymphocytes in an ACE2-independent manner (12). In addition, an *in vitro* study found that SARS-CoV-2 directly infected and replicated in inflammatory monocytes and lymphocytes, leading to apoptosis of T lymphocytes *in vitro* (13). COVID-19 patients suffering from lymphopenia almost always exhibit significant decreases in T-cell counts (14). Shima studied lymphocyte subsets in pediatric patients and found that the CD4^+^/CD8^+^T cell ratio was lower in patients with severe COVID-19 than in those with mild/moderate forms of the disease (15). The decrease in B cell counts among severe COVID-19 patients was not as consistently observed as the decrease in T cell counts (16). Another study found that two COVID-19 patients with X-linked agammaglobulinemia, a rare disorder causing a lack of mature B cells, fully recovered (17).

COVID-19 infection can result in the exhaustion of T cells. Programmed cell death protein 1 (PD-1) expression on T cells indicates exhaustion and is studied in diseases like infection, cancer, and sepsis (18). Both CD4^+^ and CD8^+^ T cells from COVID-19 patients increased the cell surface expression of PD-1 (19). In addition, SARS-CoV-2 infection can interfere with T cell expansion (20). Previous studies indicated that COVID-19 can lead to lymphopenia, with lower lymphocyte counts associated with severe infections. Further study is needed to understand the potential long-term lymphopenia from COVID-19 and its impact on future infection risk.

Another potential reason for the surge in post-pandemic infectious diseases could be the impact of the COVID-19 pandemic on overall vaccination coverage rates. In the United States, vaccine doses for children, including the MMR vaccine (including the measles, mumps, and rubella vaccine), dropped significantly during March–May 2020 due to stay-at-home orders. After these orders were lifted, vaccination rates nearly returned to pre-pandemic levels by June–September 2020 (21). In addition, during the 2020–2021 and 2021–2022 influenza seasons, influenza vaccine uptake decreased uniformly among children compared to the pre-pandemic period (22). A study found that among parents whose children missed the 2019–2020 flu vaccine, 34% were less likely and 21% were more likely to vaccinate their children for 2020–2021 due to the COVID-19 pandemic, indicating the pandemic alone wasn't enough to boost pediatric flu vaccination rates (23). While our study did not account for the prior vaccination status of the children, but existing research shows a drop in vaccination rates, including for influenza, during the pandemic. Future studies should explore vaccination trends among Chinese children during COVID-19 and their possible link to the rise in notifiable infectious diseases afterward.

Severe cases were excluded from our study. Nonetheless, prior research indicates that the immune function in children with severe COVID-19 infection is compromised compared to those with non-severe infection (11). Our study has concluded that there has been a general increase in the incidence of infectious diseases post-COVID-19, suggesting a potential rise in severe cases.

Historically, pediatric infectious diseases have threatened children's health globally, increasing morbidity and mortality rates. The emergence of COVID-19 has compounded these challenges. Prior study showed that NPI significantly reduced respiratory virus transmission in children (24), whereas our study systematically analyzed pediatric data from 2020 to 2023 to understand the COVID-19 pandemic's long-term impact on notifiable infectious diseases in children. As healthcare systems contend with the rising incidence of pediatric cases, the need for targeted interventions becomes increasingly critical.

### Limitations

While the research presents intriguing findings, it has limitations. Firstly, resource constraints and ethical issues make it difficult to gather data on pediatric notifiable infectious diseases from other hospitals, potentially introducing bias. However, this is partially mitigated by the high volume of pediatric cases at our hospital, where diagnoses and treatments follow established guidelines, ensuring our data reliably reflects disease incidence trends. Secondly, this study employs a cross-sectional design, which inherently limits the ability to establish causal relationships. Thirdly, all the cases included in our study were mild, with an absence of severe cases, thereby precluding an accurate assessment of the incidence of severe cases following the COVID-19 pandemic. Future research should conduct prospective studies with a larger sample to evaluate immune function in patients, validate current findings, and clarify the mechanisms underlying increased infectious diseases in children post-pandemic.

## Conclusion

Our research provides empirical data that establish an association between the relaxation of COVID-19 restrictions and increased pediatric infectious disease occurrence. The number of pediatric outpatient and emergency department visits and the incidence rate of notifiable infectious diseases in 2023 have markedly increased compared to the period from 2020 to 2022. Notably, the incidence of influenza has experienced the most significant increase, whereas the incidence rates of measles and mumps have remained stable.

## Data Availability

The raw data supporting the conclusions of this article will be made available by the authors, without undue reservation.
